# AutoPEWS: Automating Pediatric Early Warning Score Calculation Improves Accuracy Without Sacrificing Predictive Ability

**DOI:** 10.1097/pq9.0000000000000274

**Published:** 2020-03-25

**Authors:** Justin M Lockwood, Jacob Thomas, Sara Martin, Beth Wathen, Elizabeth Juarez-Colunga, Lisa Peters, Amanda Dempsey, Jennifer Reese

**Affiliations:** From the * University of Colorado School of Medicine, Department of Pediatrics, Section of Hospital Medicine, Aurora, Colo.; † University of Colorado, Adult and Child Consortium for Health Outcomes Research and Delivery Science (ACCORDS), Aurora, Colo.; ‡ Children’s Hospital Colorado, Aurora, Colo.

## Abstract

**Introduction:**

Pediatric early warning scores (PEWS) identify hospitalized children at risk for deterioration. Manual calculation is prone to human error. Electronic health records (EHRs) enable automated calculation, removing human error. This study’s objective was to compare the accuracy of automated EHR-based PEWS calculation (AutoPEWS) to manual calculation and evaluate the non-inferiority of AutoPEWS in predicting deterioration.

**Methods:**

We performed a retrospective cohort study inclusive of non-intensive care unit inpatients at a freestanding children’s hospital over 4.5 months in Fall 2018. AutoPEWS mapped the historical manual PEWS scoring rubric to frequently used EHR documentation. We determined accuracy by comparing the expected respiratory subset score based on the current respiratory rate to the actual respiratory score of AutoPEWS and the manual PEWS. The agreement was determined using kappa statistics. We used predicted probabilities from a generalized linear mixed model to calculate areas under the curve for each combination of scores (AutoPEWS, manual) and deterioration outcome (rapid response team activation, unplanned intensive care unit transfer, critical deterioration event). We compared the adjusted difference in areas under the curves between the scores. Non-inferiority was defined as a difference of <0.05.

**Results:**

There were 23,514 total PEWS representative of 5,384 patients. AutoPEWS respiratory scores were 99.97% accurate, while the manual PEWS respiratory scores were 86% accurate. AutoPEWS were higher overall than the manual PEWS (mean 0.65 versus 0.34). They showed a fair-to-good agreement (weighted kappa 0.42). Non-inferiority of AutoPEWS compared with the manual PEWS was demonstrated for all deterioration outcomes.

**Conclusions:**

Automation of PEWS calculation improved accuracy without sacrificing predictive ability.

## INTRODUCTION

Early warning scores aim to identify hospitalized children at risk for deterioration before care-team member awareness.^1^ Many variations of pediatric early warning scores (PEWS) exist across institutions.^2–5^ In all instances, clinical inputs (eg, vital signs and physical assessments) are assigned numerical values and combined to produce a total score purported to stratify a patient’s risk for deterioration. At our institution, we modified an existing PEWS^1^ to align with our institutional practice. It has been used on inpatient units for over a decade.

Traditionally, bedside nurses calculate PEWS by hand, and the final score is manually entered into the medical record. This method has many problems. For example, delayed or inconsistent nursing documentation may lead to delays in the calculation of an early warning score,^6,7^ undermining its ability to provide early, real-time identification of children at risk for deterioration. Additionally, manual calculation has poor interrater reliability and accuracy.^8–10^ This finding was true at our institution, as chart audits revealed frequent inaccuracies in manual PEWS calculation where the score commonly did not match the documented vitals signs used in its calculation.

Electronic health records (EHRs) are widely used in pediatric hospitals^11,12^ and offer an alternate strategy for PEWS calculation.^13^ Replacing manual calculation with computer-aided manual calculation improves early warning score accuracy.^9,10,14^ Automating PEWS calculation within an EHR further leverages technology and removes the manual calculation altogether.

This study’s objective was to compare automated EHR-based PEWS calculation (AutoPEWS) to manual PEWS calculation in score accuracy by looking at the alignment between the patient’s current respiratory rate and both the AutoPEWS and the manual PEWS respiratory subset scores. A secondary objective was to compare the ability of AutoPEWS and the manual PEWS to predict subsequent deterioration. We hypothesized that AutoPEWS would be more accurate than the manual PEWS without sacrificing predictive ability.

## METHODS

We used a retrospective, observational study design. The study was approved for conduct as non-human subject’s research by the study site’s institutional Internal Review Board as well as the Organizational Research Risk and Quality Improvement Panel (COMIRB protocol #19-1792 and ORRQIRP protocol #1806-3).

### Setting

Our institution is a quaternary care, free-standing children’s hospital. We have an onsite Pediatric Intensive Care Unit (ICU), Cardiac ICU, and Neonatal ICU but do not utilize a step-down unit, intermediate care unit, or complex care service. Within pre-specified parameters, we permit certain higher acuity interventions in non-ICU units, including but not limited to high flow nasal cannula oxygen, continuous albuterol, continuous insulin infusion, tracheostomy dependence, noninvasive positive pressure dependence, and ventilator dependence. Our institutional Code Committee oversees the PEWS improvement work.

### Intervention

Our institution has used PEWS since 2008. We adopted it from the original Brighton PEWS,^1^ which we adapted for our clinical environment to reflect patient complexity and acuity that are unique to our institution. We used the Pediatric Advanced Life Support definitions^15^ of normal vital sign ranges in our original PEWS calculation. Before this study, bedside nurses calculated the score manually using scoring rubric badge cards. They then entered the score into nursing flowsheets in our EHR. The score was calculated on all hospitalized children in non-ICU units at least every 4 hours. PEWS was initially used as a trigger for mandatory rapid response team (RRT) activation when it surpassed 4, but this trigger was removed in Fall 2017. At the time of this study, PEWS continued to be calculated on all hospitalized patients with no mandatory action based on a given score. Once manually entered into the EHR, the score was displayed prominently in the EHR in care-team facing locations as well as on the provider handoff tool.^16^ A color gradient assisted in characterizing this PEWS value into low (<3), intermediate (3–4), and high risk (>4) groups. This PEWS is referred to as the manual PEWS for the remainder of this article.

Because the primary objective of this initial study was to compare the accuracy of 2 methods of calculating PEWS, we attempted to convert our existing manual scoring rubric to automated code without making significant changes to the content of the score. We mapped the manual scoring rubric to existing, commonly used nursing documentation fields in our EHR. In doing so, we ensured that no changes to documentation practices were needed from care-team members; instead, the new automated score (AutoPEWS) would automatically recalculate with any update to clinical documentation within their existing workflow. The inputs for the original manual PEWS and the corresponding inputs for AutoPEWS are shown in Table 1.

**Table 1.**
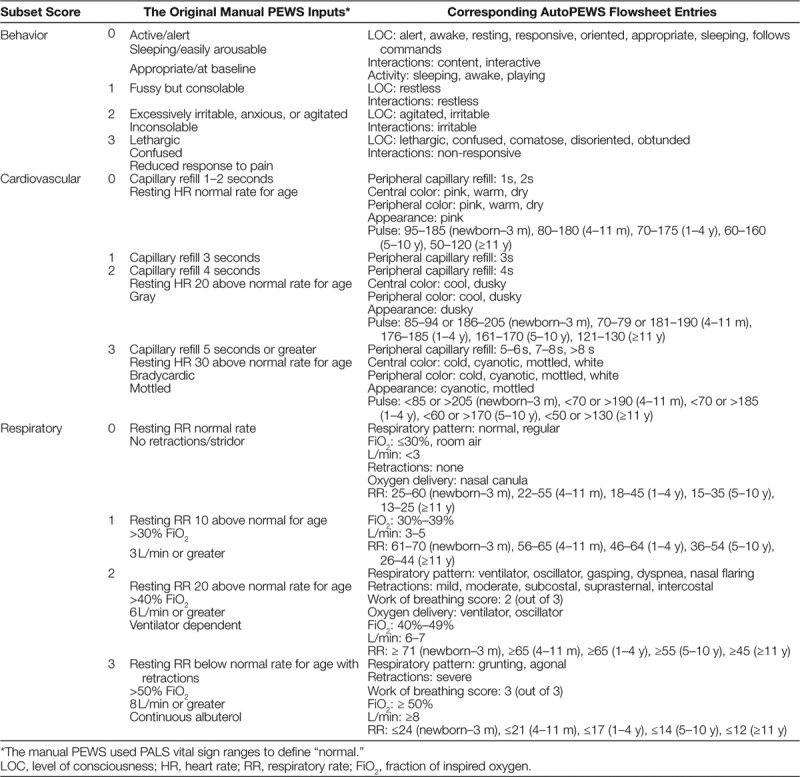
Comparison of Manual PEWS and AutoPEWS Scoring Inputs

We aligned vital sign ranges for the AutoPEWS code with the work of a separate process improvement group at our institution tasked with redefining our normal vital sign ranges in an evidence-based manner. This group used 2 published studies as a starting point for normal vital sign ranges in hospitalized children^17,18^ and then adjusted these ranges based on analysis of admissions at our institution. The included vital sign ranges are available in Table 1.

AutoPEWS continuously re-calculates whenever any single input is updated in the EHR. It pulls from documentation entered by all members of the care-team, including nurses, respiratory therapists, clinical assistants, and physicians. Neither AutoPEWS nor the manual PEWS triggered any mandatory clinical interventions during the study period. AutoPEWS was neither care-team nor patient-facing during the study period. The manual PEWS was displayed per usual practice, as described above.

### Study Population and Data Source

All patients hospitalized in non-ICU, non-psychiatry units met the inclusion criteria for this study, and we excluded no patients. The study period lasted 4.5 months in Fall 2018 (July to November). During the study period, we ran AutoPEWS silently in the background while current practice (the manual PEWS) continued. Both scores—as well as current respiratory rate and patient demographics—were collected daily at 0400 via an automated data report. We chose this time (0400) because it was near the end of both physician and nursing night shifts and corresponded with routine vital sign checks for nursing, thus optimizing the chances that the manual PEWS would be up to date at the time of data capture.

Data on deterioration outcomes were obtained directly from our institutional Code Database. The Code Database is a robust dataset maintained by our Code Committee and populated through automated reporting of all RRT/Code Team Activations supplemented by manual chart review.

### Measures of Interest

Our primary outcomes of interest were the accuracy and predictive ability of both AutoPEWS and manual PEWS. We approximated accuracy by comparing the actual AutoPEWS and manual PEWS respiratory subset scores to their expected minimum respiratory subset score correlating with the documented respiratory rate. Because a given respiratory rate is associated with a PEWS respiratory subset score (Table 1), we were able to determine the minimum expected score based on the patient’s respiratory rate at the time of data collection. We considered the actual respiratory subset score for the manual PEWS or AutoPEWS inaccurate if they were lower than the expected minimum score. We did not consider scores inaccurate if they were higher than the expected minimum score because the higher score could have been related to other PEWS inputs independent of respiratory rates such as levels of oxygen support or signs of difficulty breathing. This measure was a dichotomous variable (accurate versus not accurate).

We then tested the non-inferiority of AutoPEWS compared with the manual PEWS in detecting 4 deterioration outcomes: RRT activation, RRT or code team activation (combined), unplanned ICU transfer, and critical deterioration events (CDEs). Bonafide et al^19^ define CDEs as deterioration events leading to unplanned ICU transfer plus life-sustaining interventions (vasopressors, intubation, or positive pressure ventilation) in the subsequent 12 hours. This outcome better represents a patient’s need for critical care than unplanned ICU transfer alone.

Covariates included age, gender, admitting service, and repeat event. We dichotomized age to <1 year and 1 year and older. We collapsed admitting services based on clinical relevance. Because many patients were admitted for multiple days, we created a repeat event variable under the assumption that a patient on day 1 of hospitalization may be treated differently than the same patient better known to the care-team on a subsequent day.

### Analysis

We used descriptive statistics to evaluate the distribution of PEWS values across the study population (Table 2). Accuracy of the manual PEWS and AutoPEWS were approximated by comparing their actual respiratory subset scores to the expected minimum respiratory subset scores based on the patient’s respiratory rate at the time of data collection, as described above. The agreement was determined between the total manual PEWS and AutoPEWS—as well as each subset score—using weighted kappa statistics. We categorized kappa scores >0.75 as excellent agreement (beyond chance), scores <0.40 as poor agreement, and scores between 0.40 and 0.75 as fair-to-good agreement.^20^

**Table 2.**
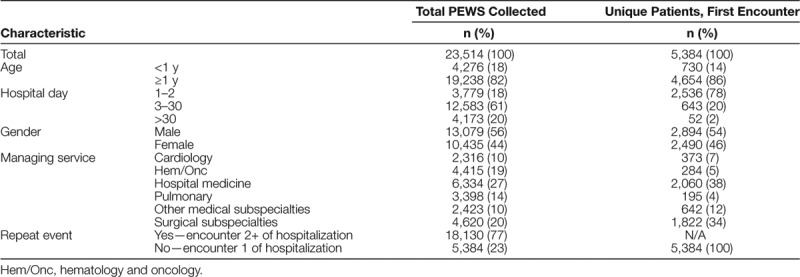
Characteristics of the Study Population

The prediction accuracy of the total manual PEWS and AutoPEWS was compared for all 4 deterioration outcomes (RRT activations, RRTs/code team activations, unplanned ICU transfers, and CDEs) via receiver operating curve (ROC) analysis. Because we collected PEWS values once daily, we paired deterioration outcomes with PEWS if the outcome occurred within the 24 hours following data collection. To control for potential confounders and repeated measures for each outcome, we identified covariates for inclusion in a generalized linear mixed model (GLIMMIX). We identified age and gender a priori due to their clinical relevance and included admitting service and repeat event based on inclusion criteria (*P* < 0.2) from the bivariable analysis. Due to sample size limitations, there was no adjustment for any covariates in CDE analysis. The predicted probabilities from GLIMMIX were used to calculate the area under the curve (AUC) for each combination of PEWS and outcome. For each outcome, the manual PEWS ROC and the AutoPEWS ROCs were compared by taking the difference in AUC. We used an inferiority margin of 0.05 to determine non-inferiority, meaning AutoPEWS would be considered non-inferior to the manual PEWS if the lower limit of the 95% confidence interval for the AUC difference between scores was >−0.05.^21,22^

Data were analyzed using SAS version 9.4 software (Cary, NC). We performed all statistical tests with a level of significance of alpha = 0.05.

## RESULTS

**Fig. 1.**
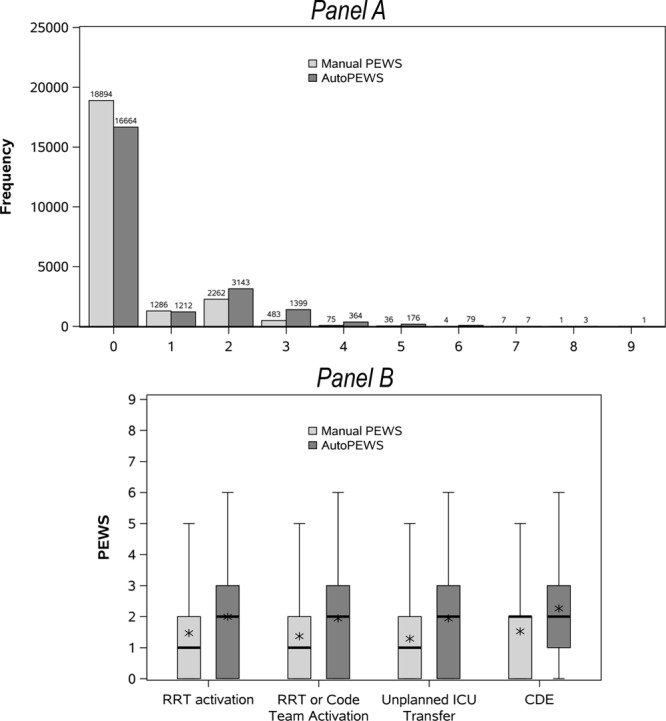
Distribution of both total manual PEWS and AutoPEWS displayed as combined histograms (A) and box and whisker plots stratified by deterioration outcome (B).

We collected 23,514 PEWS representing 5,384 unique patients (Table 2). Of the 23,514 collected PEWS, 132 (0.6%) had paired RRT activations, 85 (0.4%) had paired unplanned ICU transfer, 39 (0.2%) had paired CDEs, and 22 (0.1%) had paired code team activations. We displayed the distribution of PEWS graphically in Figure 1. AutoPEWS were higher on average than the manual PEWS (mean 0.65 versus 0.34).

### Accuracy and Agreement

AutoPEWS was 99.97% accurate, and the manual PEWS was 86% accurate to the minimum expected respiratory subset score based on the documented respiratory rate. The weighted kappa comparing the total manual PEWS and AutoPEWS was 0.42 indicating a fair-to-good agreement beyond chance. Within this comparison of AutoPEWS and the manual PEWS, the respiratory subset scores showed a fair-to-good agreement beyond chance (weighted kappa 0.59), while the behavior and cardiovascular subset scores showed poor agreement (weighted kappa 0.23 and 0.20, respectively).

### Non-inferiority

**Fig. 2.**
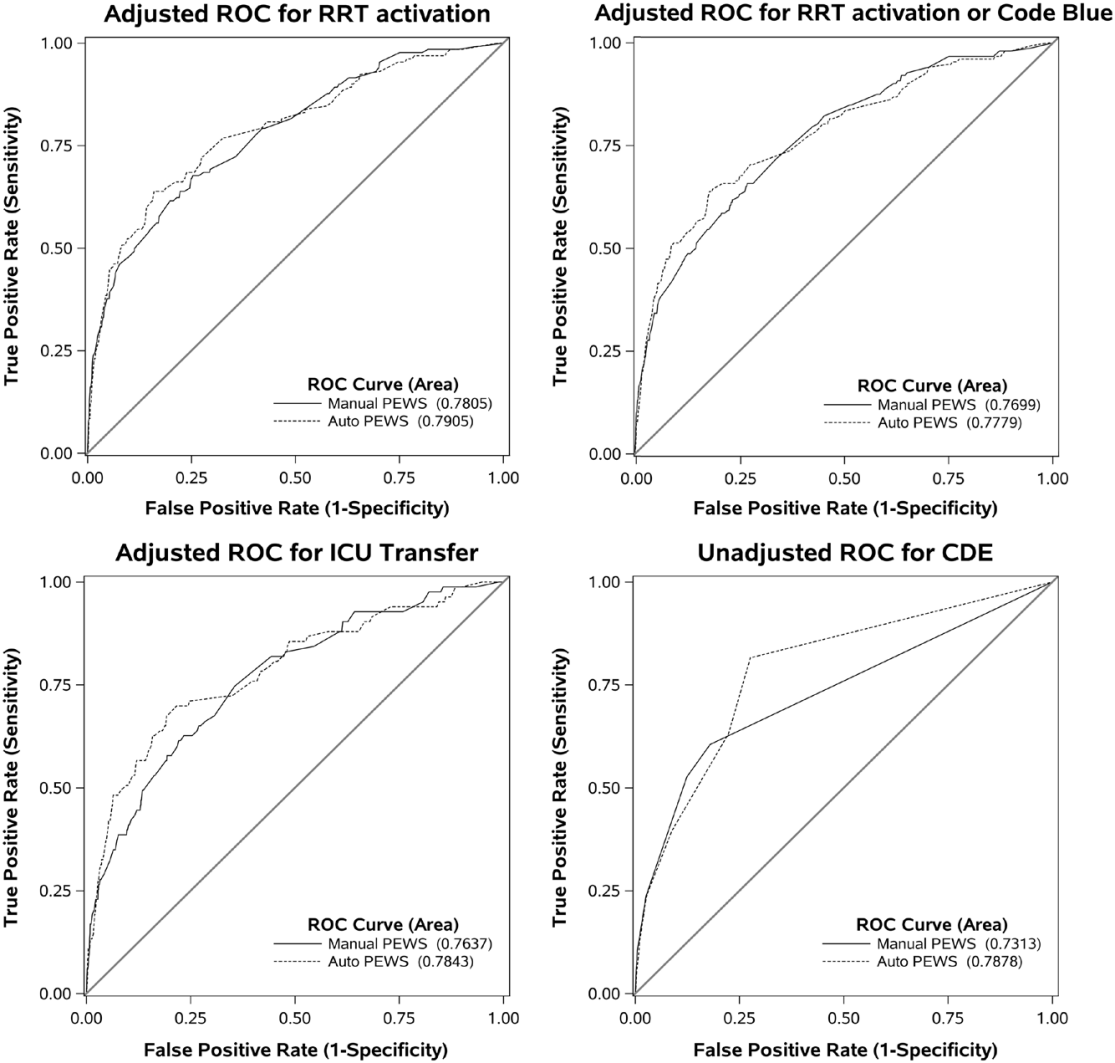
ROCs for all deterioration outcomes.

The difference in adjusted AUC (AutoPEWS—the manual PEWS) was 0.01 (95% CI −0.02 to 0.04; *P* = 0.5) for RRT activation, 0.008 (95% CI −0.02 to 0.03; *P* = 0.5) for RRT/Code Team activation, and 0.02 (95% CI −0.013 to 0.054; *P* = 0.2) for unplanned ICU transfer (Fig. 2). The difference in unadjusted AUC for CDEs was 0.06 (95% CI −0.01 to 0.13; *P* = 0.1). The lower confidence limit for the difference in AUC’s of all 4 outcomes was >−0.05, thus demonstrating non-inferiority of AutoPEWS compared with the manual PEWS for all outcomes.

## DISCUSSION

Our analysis showed an impressive improvement in PEWS respiratory subset score calculation accuracy from only 86% with the manual PEWS to nearly 100% with AutoPEWS. We hypothesize that many of the inaccuracies in the manual PEWS calculation occur in patients for whom the care-team has not yet developed concern. Because manual calculation is prone to human error, care-team members may be more likely to enter a low manual PEWS (eg, 0 or 1) without truly addressing the scoring rubric when they are not worried about a patient. This impression is supported by the lower mean total manual PEWS compared with AutoPEWS in our study population. Additionally, care-team members may be more likely to skip a calculation altogether when they are not worried about a patient, which is a possible explanation for the 2% of cases with missing manual PEWS values in our data.

The lower agreement between cardiovascular and behavior subset scores in our analysis suggests criteria in these systems may be more likely to be missed or ignored. Team members, therefore, are at risk to miss subtle changes in clinical status that may have otherwise alerted the care-team of impending deterioration earlier in the process than they observed on their own. Similarly, when care-team members are worried about a patient, they may be more likely to document a more accurate and often higher PEWS, and they may even document a higher PEWS than the rubric would suggest, aligning the score with their level of worry. The primary issue with the behavior suggested by this hypothesis is that PEWS is meant to be a surveillance score to alert care-team members before they are worried to redirect attention to an at-risk patient before the care-team would otherwise be aware. If PEWS does not begin to rise until a care-team member becomes worried about the patient—and the score then becomes more accurate—it cannot fulfill its original purpose of rising before care-team awareness. Automation of the score within the EHR solves this problem by removing the care-team member from the calculation. A rising AutoPEWS—calculated by the EHR and displayed in a front-facing manner to the care-team—may alert the team that a patient about whom they were previously not concerned may warrant closer monitoring and assessment than otherwise would have been provided.

Many institutions use PEWS as a trigger for mandatory intervention. Though several published studies have evaluated the ability of various PEWS to predict impending deterioration, few have considered whether the scores represent the intended clinical information. Our findings suggest PEWS may be frequently inaccurate when manually calculated by care-team members. As such, further caution is needed when using PEWS as a clinical decision tool to trigger interventions automatically. Additionally, steps should be taken to move toward leveraging available technologies to automate PEWS within EHRs rather than relying on manual calculation by care-team members.

Several qualitative studies have shown the value of PEWS as a tool for empowering care-team members to be assertive when concerned about a patient. Though this is an important consideration, PEWS was not created as a tool to verify that a patient is deteriorating after the care-team has become concerned, but rather as a tool to alert the care-team that a patient is at risk for deterioration before team awareness that they should be concerned. Consequently, PEWS may not be the most efficient tool for quantifying a care-team member’s “worry” and empowering him/her to speak up. We, therefore, feel the benefit of obtaining a more accurate score earlier in the process of deterioration through AutoPEWS calculation outweighs any benefit that may come from the correlation between the higher manual PEWS and care-team member worry. Other tools exist to quantify care-team member worry and facilitate assertion practices, and their implementation should be considered as part of a comprehensive care escalation system.

Of note, we believe our analysis underestimated the inaccuracy of the manual PEWS calculation by only considering the respiratory rate and respiratory subset score. There are many additional inputs included in both the manual PEWS and AutoPEWS calculations, and each input would be prone to human error and thus further inaccuracy with manual calculation. Consequently, it is most appropriate to conclude that the manual PEWS calculation was inaccurate at least 14% of the time and that the true number is likely significantly higher. Leveraging technology within our EHR to automate PEWS calculation—thus removing human error—greatly improved its accuracy to nearly 100%, which we would expect to remain true even when all inputs are evaluated.

In our accuracy analysis, the documented AutoPEWS respiratory subset score did not match our expected score in 0.03% of cases (n = 7/23,514). Because AutoPEWS should not deviate from the written code, we were surprised to find an AutoPEWS accuracy of <100%. We reviewed all 7 charts manually. In all 7 cases, we discovered the AutoPEWS calculation functioned appropriately within our EHR, and the perceived inaccuracy was due exclusively to an error in our analysis. Specifically, our analysis used patient age in months to determine normal respiratory rate ranges—and, therefore, expected respiratory subset scores—while the EHR used age in days within AutoPEWS. In all 7 cases, we would have considered AutoPEWS accurate if we calculated the expected respiratory subset score using the same age range as the EHR. Practically speaking, AutoPEWS functioned with 100% accuracy within our EHR.

Our study has additional limitations. First, the manual PEWS was visible to the care-team throughout the study period, while AutoPEWS was not. Consequently, the manual PEWS may have affected behavior and influenced our deterioration outcomes in a way that AutoPEWS could not, though there was no mandatory intervention based on a given manual PEWS. Second, part of the work in developing AutoPEWS included updating the institutionally defined “normal” vital sign ranges. As such, the expected respiratory subset score based on the respiratory rate in our accuracy analysis was not always identical between AutoPEWS and the manual PEWS, and direct statistical comparison was not possible. Third, the findings represent only a single site using a customized PEWS that is not used at other institutions to the best of our knowledge, limiting generalizability.

## CONCLUSIONS

Automated PEWS calculation within the EHR was more accurate than traditional manual calculation. Further, automated calculation was non-inferior to manual calculation in predicting clinical deterioration. When developing or improving a care escalation system, teams should strongly consider leveraging their EHR to automate PEWS calculation if not yet done.

## DISCLOSURE

The authors have no financial interest to declare in relation to the content of this article.
